# 3D mosquito screens to create window double screen traps for mosquito control

**DOI:** 10.1186/s13071-017-2322-2

**Published:** 2017-08-29

**Authors:** Ayman Khattab, Kaisa Jylhä, Tomi Hakala, Mikko Aalto, Robert Malima, William Kisinza, Markku Honkala, Pertti Nousiainen, Seppo Meri

**Affiliations:** 1 Research Program Unit, Immunobiology Research Program and Department of Bacteriology and Immunology, Haartman Institute, University of Helsinki, Haartmaninkatu 3, FIN-00014 Helsinki, Finland; 2 Department of Nucleic Acid Research, Genetic Engineering and Biotechnology Research Institute, City of Scientific Research and Technological Applications, Alexandria, Egypt; 3 Department of Materials Science, Tampere University of Technology, P.O. Box 589, 33101 Tampere, Finland; 4 Bosaso General Hospital, Bosaso, Somalia; 5 National Institute for Medical Research, Amani Medical Research Centre, Muheza, Tanzania; 6 Helsinki University Central Hospital, Haartmaninkatu, FIN-00029 Helsinki, Finland

**Keywords:** Mosquito, Control, 3D-screen, Window, Trap

## Abstract

**Background:**

Mosquitoes are vectors for many diseases such as malaria. Insecticide-treated bed nets and indoor residual spraying of insecticides are the principal malaria vector control tools used to prevent malaria in the tropics. Other interventions aim at reducing man-vector contact. For example, house screening provides additive or synergistic effects to other implemented measures. We used commercial screen materials made of polyester, polyethylene or polypropylene to design novel mosquito screens that provide remarkable additional benefits to those commonly used in house screening. The novel design is based on a double screen setup made of a screen with 3D geometric structures parallel to a commercial mosquito screen creating a trap between the two screens. Owing to the design of the 3D screen, mosquitoes can penetrate the 3D screen from one side but cannot return through the other side, making it a unidirectional mosquito screen. Therefore, the mosquitoes are trapped inside the double screen system. The permissiveness of both sides of the 3D screens for mosquitoes to pass through was tested in a wind tunnel using the insectary strain of *Anopheles stephensi*.

**Results:**

Among twenty-five tested 3D screen designs, three designs from the cone, prism, or cylinder design groups were the most efficient in acting as unidirectional mosquito screens. The three cone-, prism-, and cylinder-based screens allowed, on average, 92, 75 and 64% of *Anopheles stephensi* mosquitoes released into the wind tunnel to penetrate the permissive side and 0, 0 and 6% of mosquitoes to escape through the non-permissive side, respectively.

**Conclusions:**

A cone-based 3D screen fulfilled the study objective. It allowed capturing 92% of mosquitoes within the double screen setup inside the wind tunnel and blocked 100% from escaping. Thus, the cone-based screen effectively acted as a unidirectional mosquito screen. This 3D screen-based trap design could therefore be used in house screening as a means of avoiding infective bites and reducing mosquito population size.

**Electronic supplementary material:**

The online version of this article (doi:10.1186/s13071-017-2322-2) contains supplementary material, which is available to authorized users.

## Background

Malaria is a deadly disease that is endemic in a number of tropical countries [1, 2]. It is transmitted by the bite of several species of the genus *Anopheles* [3]. Malaria mosquitoes have preferential feeding habits as some tend to favor feeding indoors, such as the African vectors *An*. *gambiae* (*sensu stricto*) and *An*. *funestus* (*s.s.*) [4, 5] and the Asian vector *An*. *stephensi* (*s.s*.) [6], while others feed outdoors and indoors (ambivalent feeding behavior), such as *An*. *arabiensis* [7]. Minimizing man-mosquito contact is considered one of the main and most successful means of reducing the burden of malaria in endemic areas [8]. This has been achieved by using the insecticide-treated bed nets (ITNs), which physically protect individuals sleeping under the net. ITNs, when in good condition and well treated, also protect individuals sleeping outside them [1]. ITNs, indoor residual spraying of insecticides (IRS) and artemisinin-based combination therapies (ACTs) are the major malaria intervention tools currently recommended by WHO and included in malaria control campaigns [1]. The extensive use of malaria control measures, based on insecticides and therapeutic drugs, could lead to the emergence of vector resistance to insecticides and drug-resistant parasites [9]. Thus, additional supportive malaria control measures are needed to ease the pressure on the existing ones. An efficient and environment friendly method, no or less insecticide-dependent method, would be a better match. House screening, which has long been used in developed countries, mostly to keep nuisance mosquitoes away, was associated with protection against malaria when implemented in mosquito control studies in malaria endemic areas [10, 11]. A recent study by Kirby et al. [12] measured the clinical outcomes of house screening in an African setting and found that window and door screens and closed eaves halved the prevalence of anemia in children. In another recent study by Diabate et al. [13], house screening and mosquito trapping were combined in one tool to control mosquito populations. This intervention significantly reduced the number of mosquitoes in houses and killed the trapped mosquitoes [13]. Despite reports on the success of house screening in field studies house screening use is still sparse, probably due to installation and maintenance costs. We developed a novel screen design for house screening. The study was based on a systematic search for structures made of polyester, glass fiber reinforced polyester, polyethylene, polypropylene, or cellulose acetate that would allow unidirectional passage of mosquitoes. Screens made of these structures could be added in front of the traditional mosquito screen to create a window double screen trap. While preserving the benefit and concept of house screening, such structures would also allow mosquito trapping when used in a double screen setup. Screens designed and tested throughout the study were made of 3D geometric structures (cylinders, filaments, prisms and cones) evenly distributed on a commercial mosquito screen. The 3D structures would allow mosquitoes to pass through one side of the screen but not the other. Using traditional handicrafts, a set of 3D screens with cone, cylinder, filament, and prism-based structures were designed and investigated in a wind tunnel for their ability to act as a unidirectional mosquito screen in a double screen setup.

## Methods

### Mosquito rearing

*Anopheles stephensi* (Sind-Kasur Nijmegen strain) were maintained in 20 × 20 × 20 cm gauze cages at 28 °C, 80 ± 5% relative humidity, and a photo-scotophase of 12:12 with the light phase from 22.00–10.00 h and the dark phase from 10.00–22.00 h to allow running the experiments during the host-seeking active hours of the mosquitoes. The mosquitoes had access to a 5% sucrose solution on a cotton pad. The larvae were reared in tap water on plastic trays and fed daily with Tetramin® fish food (Melle, Germany). Pupae were collected daily and placed in adult cages for emergence. Adult female mosquitoes were regularly fed a blood meal that contained 1:1 human erythrocytes and human serum using the glass membrane feeder for maintaining the mosquito rearing cycle (Finnish Red Cross, Helsinki, Finland).

### Screen materials and designs

**Fig. 1:**
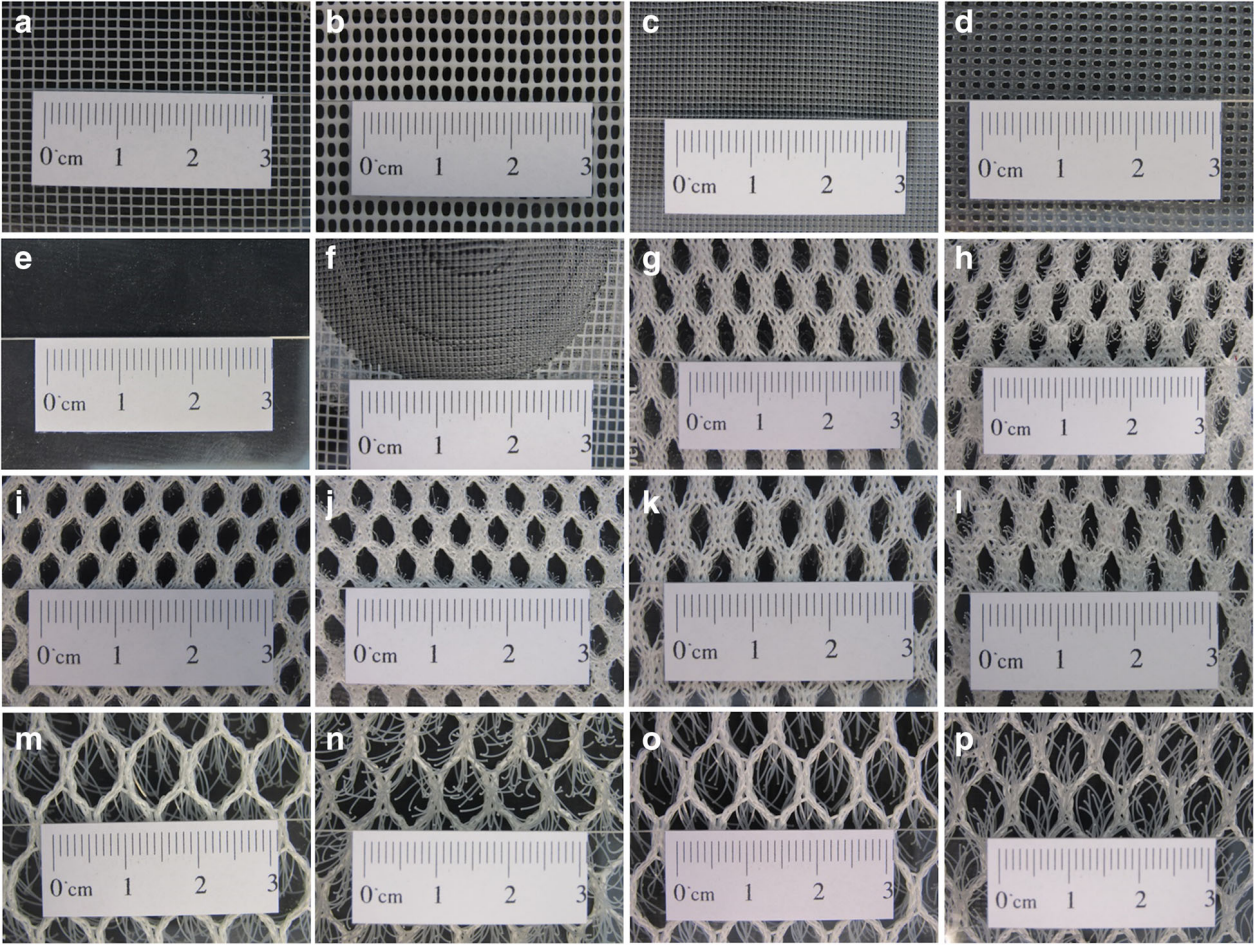
Screen materials. **a**-**d** B1w, B2w, XN4900 and XN3019 flat screens, respectively. **e** Cellulose acetate transparent film. **f** A screen made of B1w and XN4900. **g** and **h**, **i** and **j**, **k** and **l**, **m** and **n**, **o** and **p**: the two sides of the filament screens S4, S5, S6, S7 and S8, respectively

Screen materials used throughout the study are listed in Table 1. Briefly, screens were made of either glass fiber reinforced polyester (screen code: B1w), polyester (screen codes: S4, S5, S6, S7 and S8), polyethylene (screen code: B2w), polypropylene (screen codes: XN4900 and XN3019), or cellulose acetate (screen code: Film). The screens were also categorized into three groups: flat screens (B1w, B2w, XN4900 and XN3019) (Fig. 1a-d), single-sided filament screens (Fig. 1g-p), and transparent films (Fig. 1e). Twenty-five 3D screen designs (19 × 19 cm), detailed properties of which are described in Table 2, were made of the abovementioned screen materials to function as unidirectional mosquito screens. Briefly, the 3D screen designs had 3D geometric structures, cylinders, protruding filaments, prisms, or cones evenly distributed on a flat screen made of the same screening material except for a single case in which two screen materials (B1w and XN4900) were used to create a 3D screen design (Fig. 1f). The cylinder-based screens each had 36 cylinders that were devoid of the two bases to allow mosquito passage. The cone-based screen had either 4, 6, or 16 cones, also devoid of bases. The cones apices were trimmed to create 5 mm diameter pores. The prism-based screens had either 2 or 3 prisms, the bases of which were absent, and the protruding edges had either two open slits or pores, details are described in Table 2. The filament-based screens were made of 3D spacer mesh fabrics made by Baltex (Ilkeston, UK). Spacer fabric image is shown in Additional file 1: Figure S1. A cut through the vertical yarns that connects the two horizontal knitted faces of the spacer fabrics created filament-based screens. Thus, from one spacer fabric, we obtained up to two filament-based screens. The length of the filaments was dependent on both the point at which the cuts were made and the thickness of the spacer fabric. Three different spacer fabrics were used to create five different filament-based screens with variable filament length and mesh size (Table 2). The cylinder-, filament-, prism-, and cone-based screens were given the screen codes Cyl1–3, S4–8, W1–5, and C01-C12, respectively. The 3D structures were oriented on the screens to allow mosquitoes to pass through only one side of the screen design, referred to as the permissive side of the screen. The other side of the screen was designed to block mosquito penetration and referred to as the non-permissive side. Higher resolution images of the individual screens used throughout the study are provided in the Additional file 2: Figure S2.

**Table 1:** Screen materials and commercial sources

Screen material code	Screen material	Source
B1w	Glass fiber reinforced polyester	Local hardware store
B2w	Polyethylene	Local hardware store
S4	Polyester	BALTEZ, Derbyshire, UK
S5	Polyester	BALTEZ, Derbyshire, UK
S6	Polyester	BALTEZ, Derbyshire, UK
S7	Polyester	BALTEZ, Derbyshire, UK
S8	Polyester	BALTEZ, Derbyshire, UK
XN4900	Polypropylene	Industrial Netting, Minneapolis, USA
XN3019	Polypropylene	Industrial Netting, Minneapolis, USA
Film	Cellulose acetate	Local hardware store

**Table 2:** Structural properties of the 3D screens

		3D structure			
3D Screen type and code	Screen material	Height (mm)	Base diameter, area or mesh size (mm)	Tip diameter or slit area (W × H) (mm)	Number of 3D structure units
Cylinder-based					
Cyl1	B1w	25	12	12	36
Cyl2	B1w	25	9	9	36
Cyl3	B1w	20	7	7	36
Filament-based					
S4	S4	9	(6 × 11)^a^	(0.2)^e^	numerous
S5	S5	14	(6 × 11)^a^	(0.2)^e^	numerous
S6	S6	4	(3 × 5)^a^	(0.1)^e^	numerous
S7	S7	6	(3 × 5)^a^	(0.1)^e^	numerous
S8	S8	2	(4 × 5)^a^	(0.1)^e^	numerous
Prism-based					
W1	B2w	35	(155 × 30; 155 × 40; 155 × 50)^b^	(60 × 10)^f^	3
W2	B1w	20	(155 × 65)^c^	6^g^	2
W3	B2w	35	(150 × 40)^c^	(55 × 4; 50 × 4)^f^	2
W4	B2w	25	(150 × 40)^b^	4^h^	3
W5	B2w	40	(90 × 30)^c,d^	(40 × 3)^f^	3
Cone-based					
C01	B1w	40	35	5	4
C02	B1w	40	35	5	4
C03	B1w	40	40	5	6
C04	B1w	30	40	5	4
C05	B1w	30	40	5	6
C06	B1w	20	40	5	4
C07	B1w	20	40	5	6
C08	B1w	15	22	5	16
C09	XN4900	20	40	5	4
C10	B1w & XN4900	20	40	5	4
C11	XN3019	20	40	5	4
C12	Film	23	40	5	4

^a^Mesh

^b^Three triangular prisms

^c^Two triangular prisms

^d^Two right triangular angle prisms

^e^Filament diameter

^f^Two slits

^g^Diameter of eleven pores

^h^Diameter of thirteen pores

### Wind tunnel

**Fig. 2:**
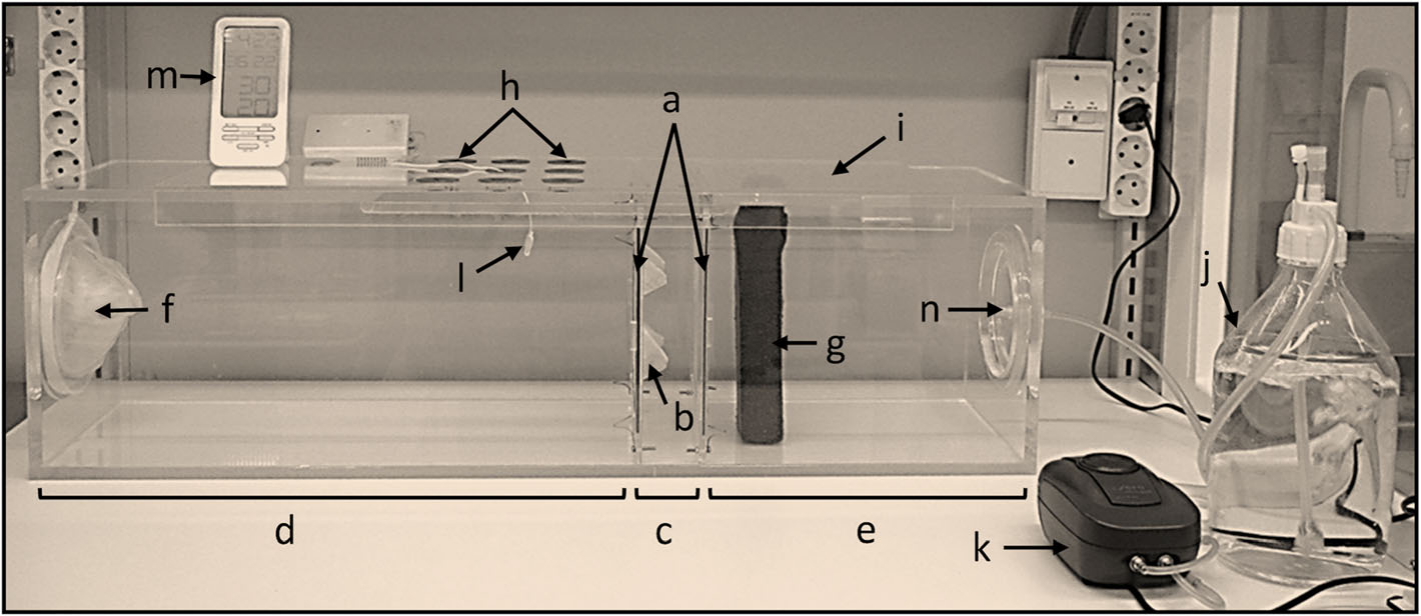
A cuboid-shaped wind tunnel (90 × 26 × 26 cm). **a** Partition frames. **b** 19 × 19 cm 3D screen. **c** Mosquito double screen trap. **d** Mosquito compartment. **e** Lure compartment. **f** Mosquito release inlet leading to the mosquito compartment. **g** Mosquito lure composed of a flat polystyrene flask with a surface area of 150 cm2 filled with warm water (40 °C) and enclosed in a worn sock. **h** Vents for releasing mosquitoes into the double screen trap. **i** Sliding lid. **j** Warm (40 °C) water bottle. **k** Air pump to push warm air into the test tunnel through warm water. **l** Temperature and humidity sensor. **m** Temperature and humidity display. **n** Warm air inlet

A wind tunnel (L × W × H; 90 × 26 × 26 cm, Fig. 2) was designed to accommodate partition frames (Fig. 2a) to hold 19 × 19 cm screens (Fig. 2b). A double screen setup was made of two parallel partition frames to hold two screens, separated by 5 cm, to form a mosquito trap within the wind tunnel (Fig. 2c). The double screen partitions divided the test tunnel into 30 and 54 cm long compartments (Fig. 2d, e). The permissive side of the 3D screen was always facing the 54 cm compartment (Fig. 2d), while the non-permissive side was facing the trap (Fig. 2c). Temperature and humidity ranging from 26 to 28 °C and 50–80%, were maintained inside the wind tunnel. This was achieved by passing the air, 8 cm/s, through a bottle containing 2 l of 40 °C water before pumping it into the tunnel. Pumping humidified air into the wind tunnel started 30 min before each experiment and continued throughout the one-hour long experiment. Temperature and humidity were monitored using a wired sensor connected to RF transmitter/receiver modules (Model: K0931, Nexus Industrial Design Ltd., Hong Kong) (Fig. 2m, n) inserted in the center of the test tunnel through a vent located on the roof of the tunnel. Mosquito host-seeking behavior inside the wind tunnel was stimulated by making use of the volatiles emanating from a human worn sock [14]. Pre-run experiments using a perforated mosquito screen in place of the 3D screen showed that worn socks alone were not sufficient for attracting most the mosquitoes to enter the double screen trap. Combing worn sock volatiles with a warm object, however, attracted on average 97% to enter the trap in four different independent experiments. Therefore, the experimental lure setup was composed of a flat polystyrene flask with a surface area of 150 cm^2^ filled with 40 °C water and enclosed in a worn sock (Fig. 2g). An adult male volunteer wore the cotton socks for an 8 h workday before each experiment. Worn socks were stored in the fridge in sealed plastic bags until the following day. Each worn sock was used only once. The same volunteer contributed worn socks for the entire study.

### Testing the permissiveness of the 3D screens

**Fig. 3:**
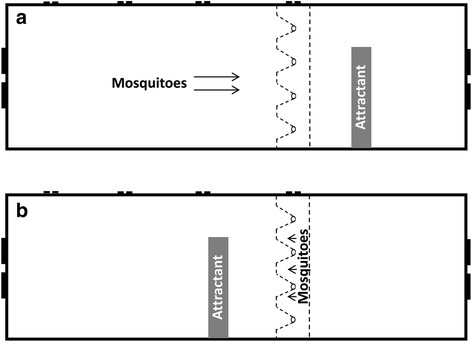
Diagram of the test tunnel experimental setup. **a** The location of the released mosquitoes and the lure when the permissive side of the 3D screens was tested. **b** The location of the released mosquitoes and the lure when the non-permissive side of the 3D screens was tested

To test the permissiveness of the 3D screen, mosquitoes were sugar starved for 12 h prior to the experiment and 40–60 of 1-week old female mosquitoes were released into the 54 cm compartment (Figs. 2d and 3a) through a sleeved opening at one end of the wind tunnel (Fig. 2f). The lure was placed in the 30 cm compartment (Fig. 2e), 4 cm away from the flat screen (B1w) side of the double screen setup. The blocking efficiency of the non-permissive side was tested by releasing mosquitoes inside the double screen trap (Figs. 2c and 3b) through a vent on the sliding lid (Fig. 2i) at the top the wind tunnel. The lure was placed in the 54 cm compartment (Fig. 2d), 4 cm away from the double screen. Experiments were done twice for each side (the permissive and the non-permissive) of the 3D screen on two different days using two different mosquito generations. Only two experiments were run per day. The inner surface of the wind tunnel was wiped with a wet towel and placed under a laboratory hood for 30 min before the next experiment was done.

### Data collection and analysis

Mosquitoes that migrated through the 3D screen from either the 54 cm compartment to the double screen trap or from the trap to the 54 cm compartment (Fig. 2d), depending on the experimental setup, were counted after each 1 h long experiment. Data are presented in Tables 3 and 4 as the number of mosquitoes released into the wind tunnel that passed the screen, and the percentage that passed the screen. The mean number of mosquitoes released into the wind tunnel that passed the screen, the mean percentage that passed the screen, and the 95% confidence interval of the percentage mean were also presented for the two experimental repetitions. Throughout the text the performance of each screen is presented as mean percent, mean passed/mean released. Statistical analysis was performed using the JMP software, version 11.0 (SAS Institute Inc., Cary, NC, USA). Screen performance (SP) index, presented in Table 4, accounting for the performance of the permissive and the non-permissive sides of the screens was calculated using the formula: $\mathrm{SP}\ \mathrm{index}=\frac{\mathrm{PENT}\ \left(100-\mathrm{PESCT}\right)}{100}$, where PENT stands for “mean percent of mosquitoes entered the trap through the permissive side” and PESCT stands for “mean percent of mosquitoes escaped the trap through the non-permissive side”. The higher the SP index (maximum 100) the better the performance of the 3D screen.

**Table 3:** Results summary of experiments done to test the permissive side of the 3D screens

	Experiment 1		Experiment 2					
	Number of mosquitoes		Number of mosquitoes		Mean^a^ number of mosquitoes			
3D screen type	Released into the wind tunnel	Passed through the screen (%)	Released into the wind tunnel	Passed through the screen (%)	Released into the wind tunnel	Passed through the screen	Mean^a^ percent passed	95% confidence interval
Cylinder-based								
Cyl1	60	34 (57)	56	29 (52)	58	32	54	23–85
Cyl2	66	40 (61)	61	41 (67)	64	41	64	22–106
Cyl3	54	10 (19)	49	11 (22)	52	11	20	-4–45
Filament-based								
S4	57	50 (88)	59	48 (81)	58	49	85	44–125
S5	57	36 (63)	52	34 (65)	55	35	64	50–78
S6	41	11 (27)	63	15 (24)	52	13	25	6–45
S7	39	3 (8)	63	7 (11)	51	5	9	-12–31
S8	43	7 (16)	49	10 (20)	46	9	18	-8–45
Prism-based								
W1	49	42 (86)	49	44 (90)	49	43	88	62–114
W2	60	42 (70)	61	39 (64)	61	41	67	28–106
W3	42	11 (26)	54	11 (20)	48	11	23	-14–60
W4	40	16 (40)	48	16 (33)	44	16	37	-6–79
W5	51	36 (71)	61	48 (79)	56	42	75	23–126
Cone-based								
C01	56	43 (77)	61	49 (80)	59	46	79	56–101
C02	53	26 (49)	58	32 (55)	56	29	52	13–91
C03	61	33 (54)	68	40 (59)	65	37	56	26–86
C04	48	45 (94)	58	52 (90)	53	49	92	66–118
C05	62	57 (92)	63	51 (81)	63	54	86	17–156
C06	59	42 (71)	46	35 (76)	53	39	74	43–105
C07	36	28 (78)	64	46 (72)	50	37	75	37–112
C08	80	57 (71)	42	31 (74)	61	44	73	56–89
C09	45	14 (31)	52	19 (37)	49	17	34	-1–68
C10	60	26 (43)	58	26 (45)	59	26	44	35–54
C11	47	28 (50)	51	26 (51)	49	27	55	1–110
C12	55	12 (22)	60	11 (18)	58	12	20	-2–42

^a^Means and percentages rounded to the nearest integer

**Table 4:** Calculated screen performance index and results summary of experiments done to test the non-permissive side of the 3D screens

	Experiment 1		Experiment 2						
	Number of mosquitoes		Number of mosquitoes		Mean^a^ number of mosquitoes				
3D screen type	Released into the wind tunnel	Passed through the screen (%)	Released into the wind tunnel	Passed through the screen	Released into the wind tunnel	Passed through the screen	Mean^a^ percent passed	95% confidence interval	Screen performance index (SP index)
Cylinder-based									
Cyl1	41	14 (34)	56	14 (25)	49	14	30	-29–88	38
Cyl2	57	2 (4)	53	5 (9)	55	4	6	-31–44	60
Cyl3	59	1 (2)	53	0 (0)	56	1 (0.5)^a^	1	-10–12	20
Filament-based									
S4	46	25 (54)	52	31 (60)	49	28	57	24–90	36
S5	69	41 (59)	62	31 (50)	66	36	55	-5–115	29
S6	61	20 (33)	45	17 (38)	53	19	35	4–67	16
S7	41	8 (20)	48	15 (31)	45	12	25	-49–100	7
S8	40	16 (40)	46	21 (46)	43	19	43	7–79	10
Prism-based									
W1	42	19 (45)	64	32 (50)	53	26	48	17–78	46
W2	58	5 (9)	57	4 (7)	58	5	8	-2–18	62
W3	53	1 (2)	44	0 (0)	49	1 (0.5)^a^	1	-11–13	23
W4	43	4 (9)	58	1 (2)	51	3	6	-43–54	35
W5	48	0 (0)	45	0 (0)	47	0	0	0	75
Cone-based									
C01	55	0 (0)	60	0 (0)	58	0	0	0	79
C02	47	0 (0)	60	0 (0)	54	0	0	0	52
C03	43	0 (0)	53	0 (0)	48	0	0	0	56
C04	58	0 (0)	45	0 (0)	52	0	0	0	92
C05	56	0 (0)	48	0 (0)	52	0	0	0	86
C06	62	0 (0)	64	0 (0)	63	0	0	0	74
C07	42	0 (0)	40	0 (0)	41	0	0	0	75
C08	54	0 (0)	41	0 (0)	48	0	0	0	73
C09	56	0 (0)	63	0 (0)	60	0	0	0	34
C10	48	0 (0)	47	0 (0)	48	0	0	0	44
C11	50	0 (0)	51	0 (0)	51	0	0	0	55
C12	62	0 (0)	43	0 (0)	53	0	0	0	20

^a^Mean rounded to one decimal place. All other means and percentages rounded to the nearest integer

## Results

**Fig. 4:**
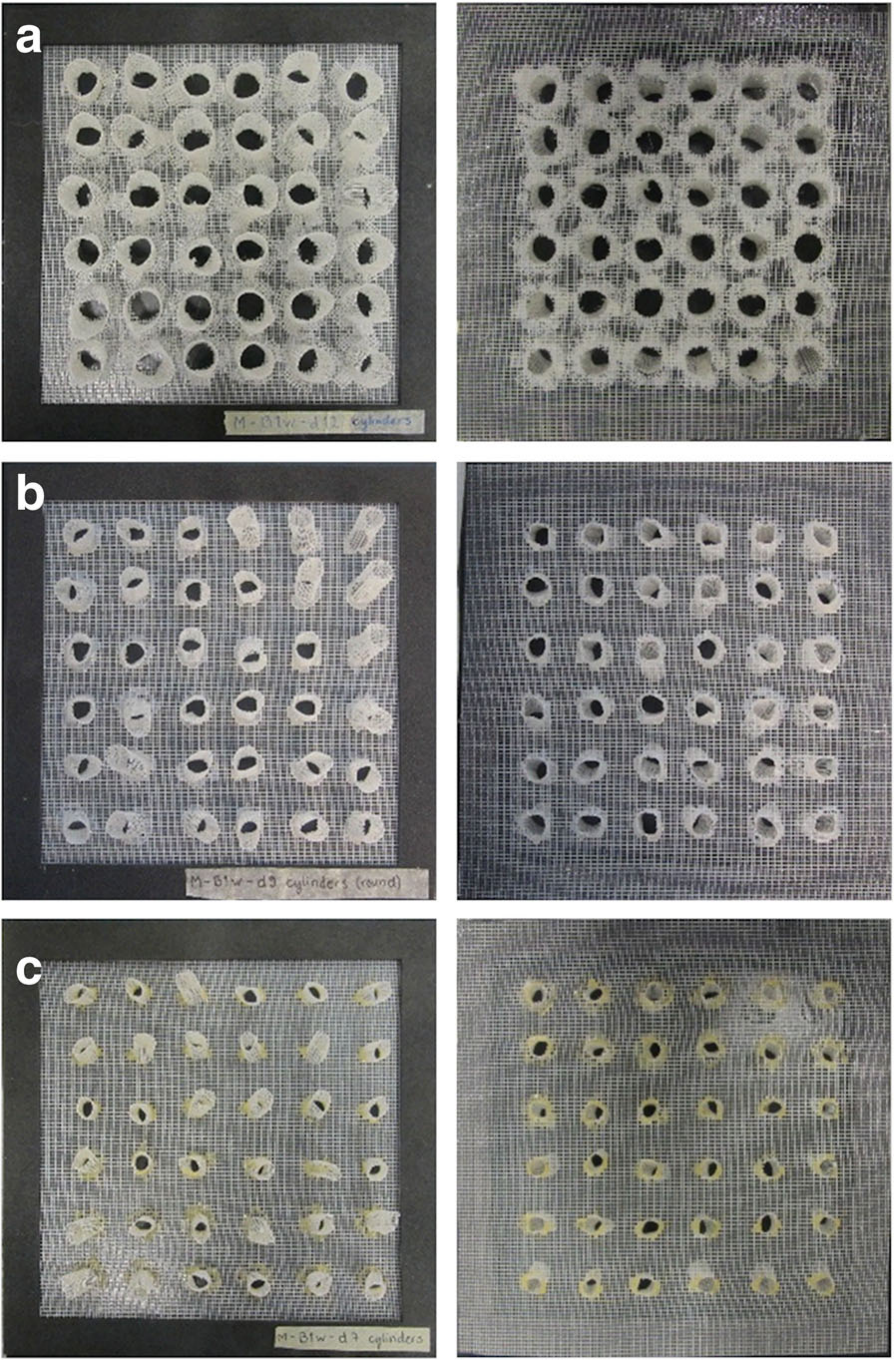
Cylinder-based 3D screens. The *left* and *right* panels show the non-permissive and permissive sides of the screens, respectively. **a**-**c** Cyl1, Cyl2 and Cyl3 screens with 12, 9 and 7 mm diameter cylinders, respectively

Three cylinder-based 3D screens, Cyl1, Cyl2, and Cyl3, (Fig. 4a-c) were tested for their performance as unidirectional screens. Detailed physical properties of the 3D screens are presented in Table 2. The highest permissiveness for a permissive side (the higher the better) of a screen was reported for Cyl2 (64%, 41/64) (Table 3) while the lowest for a non-permissive side (the lower the better) was reported for Cyl3 (1%, 0.5/56) (Table 4). The calculated SP index, presented in Table 4, which accounted for both the performance of the permissive side and the non-permissive side, assigned Cyl2 as the best cylinder-based screen with a SP index of 60, while the SP index was 38 for Cyl1 and 20 for Cyl2.

**Fig. 5:**
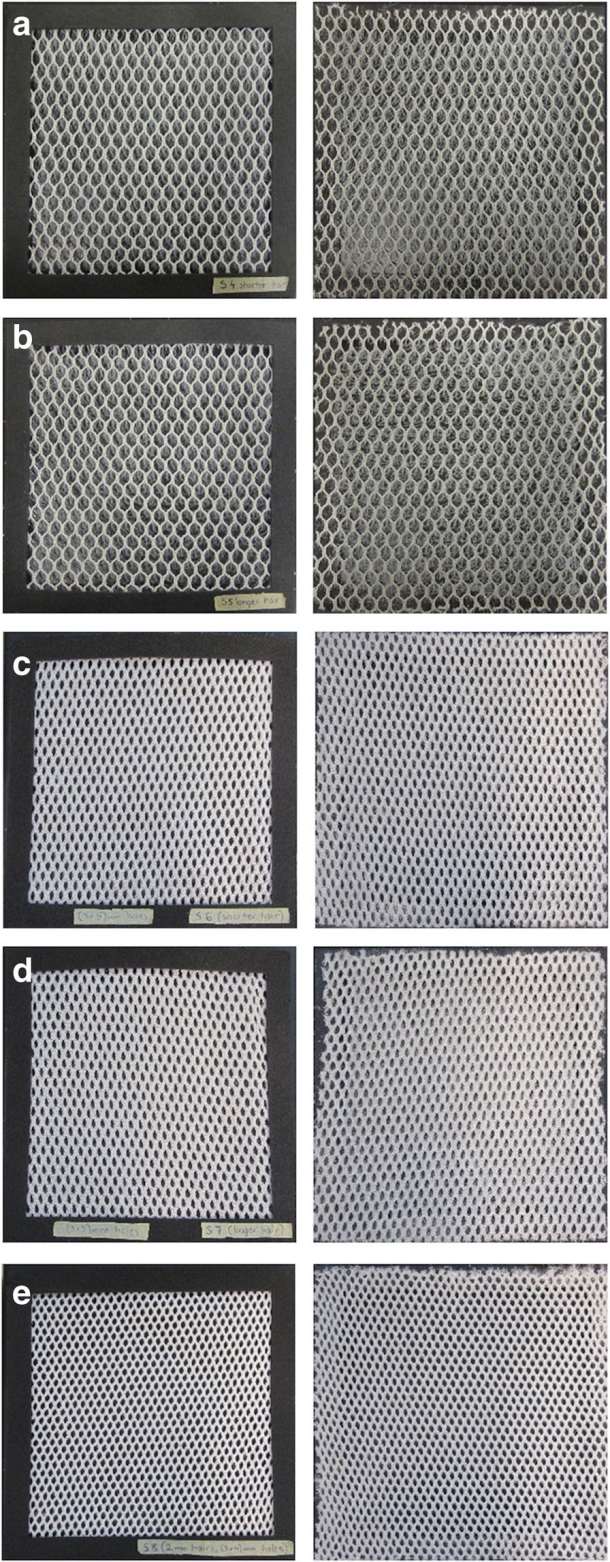
Filament-based 3D screens. The *left* and *right* panels show the permissive and non-permissive sides of the screens, respectively. **a** S4 screen with a 9 mm long filament and 6 × 11 mm mesh. **b** S5 screen with a 14 mm long filament and 6 × 11 mm mesh. **c** S6 screen with a 4 mm long filament and 3 × 5 mm mesh. **d** S7 screen with a 6 mm long filament and 3 × 5 mm mesh. **e** S8 screen with a 2 mm long filament and 3 × 4 mm mesh

Five filament-based screens, S4, S5, S6, S7 and S8 (Fig. 5a-e), with different mesh size and filament length (details in Table 2), were tested for their performance as unidirectional screens. The permissive and the non-permissive sides of the S4 screen showed the best (85%, 49/58) (Table 3) and the poorest (57%, 28/49) (Table 4) performance, respectively. On the other hand, the S7 screen presented an opposite performance pattern, as its permissive side showed the poorest (9%, 5/51) performance, while its non-permissive side showed the best (25%, 12/45) performance among the filament-based screens. The best performing filament-based screen according to the calculated SP index (Table 4), however, was S4 (with a SP index of 36) and the lowest was S7 (with a SP index of 7); the lowest reported SP index in the whole study.

**Fig. 6:**
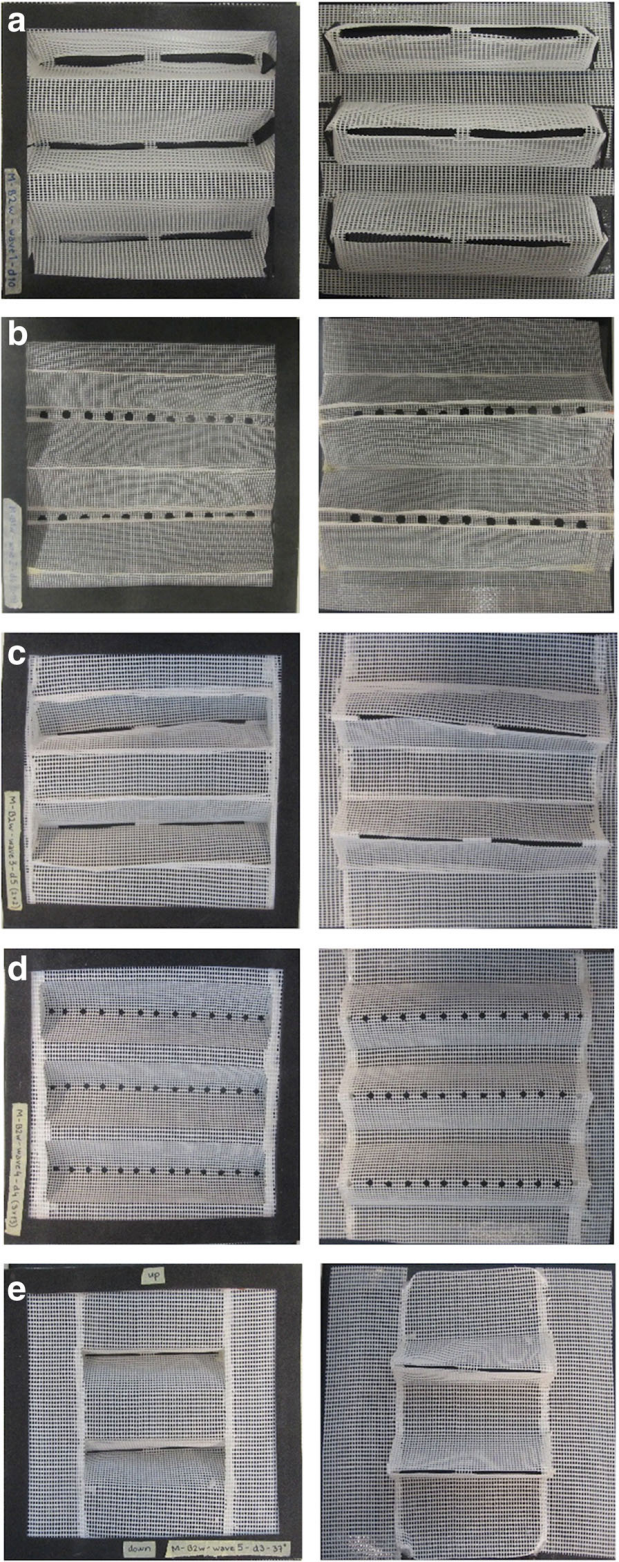
Prism-based screens. The *left* and *right* panels show the permissive and non-permissive sides of the screens, respectively. **a**-**d** screens based on triangular prisms. **e** Screen based on right triangular prisms. **a** and **c**-**e** screens were made of B2w screening material, **b** screen was made of B1w screening material. Prisms on **a**-**c** screens had 16 cm widths, while those on **e** had 10 cm widths. The exposed edges of **a** and **c** screen prisms had two slits with a length of 5–6 cm each separated by an uncut part of about 1 cm. **a** and **c** prisms had 10 and 5 mm wide slits, respectively. **e** Screen prisms had two slits with a length and width of 10 cm and 5 mm, respectively. Exposed edges of **b** and **d** had 11 pores with a 6 mm diameter and 13 pores with a 4 mm diameter, respectively. Prisms on **b** had two 4 mm long skirts along the exposed edge length creating an arc-shaped edge enclosing the pores at its deepest point. **a** and **d** screens had 3 prisms whereas **b**, **c** and **e** had only 2 prisms. The 3 prisms on **a** had 3 different base widths of 3, 4 and 5 cm from top to bottom

The third 3D screen design group was based on either triangular prisms (W1, W2, W3 and W4, Fig. 6a-d) or right triangular prisms (W5, Fig. 6e). The W1 prism-based screen had the best permissive side performance (88%, 43/49) (Table 3) and the poorest non-permissive side performance (48%, 26/53) (Table 4). The W5 screen had the best second permissive side performance (75%, 42/56), however, showed the best non-permissive side performance (0%, 0/47). The highest SP index within the prism-based screen group was 75 and was calculated for W5.

**Fig. 7:**
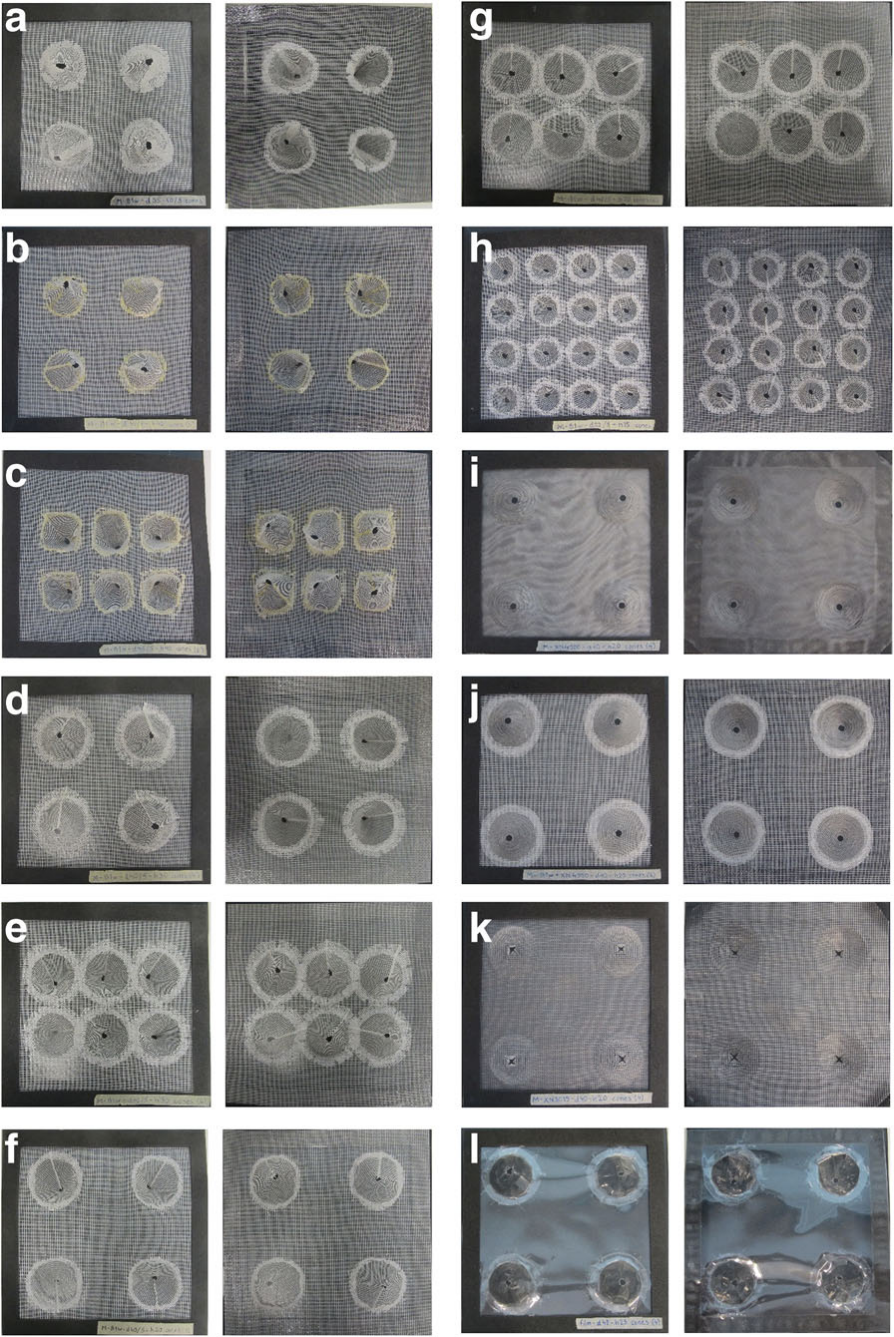
Cone-based screens. The *left* and *right* panels show the non-permissive and permissive sides of the screens, respectively. **a-l** C01-C12 cone-based screens. **a**, **b**, **d** and **i-l** Screens with 4 cones. **c**, **e** and **g** Screens with 6 cones. **h** A screen with 16 cones. **d-l** Screens with a 40 mm cone base diameters. **b** and **c** Screens with 35 mm cone base diameters. **h** A screen with a 22 mm cone base diameter. **a**-**c** Screens with 40 mm cone height diameter. **d** and **e** Screens with 30 mm cone height diameter. **f**, **g**, **i**, **j**, and **K** Screens with 20 mm cone height diameter. **h** and **l** Screen with 15 and 23 mm cone height diameter, respectively. Screen with 2 cones having 40 mm cone base diameters and 2 cones having 35 mm cone base diameters. The apexes of the cones were truncated to create a pore with a 5 mm diameter

**Fig. 8:**
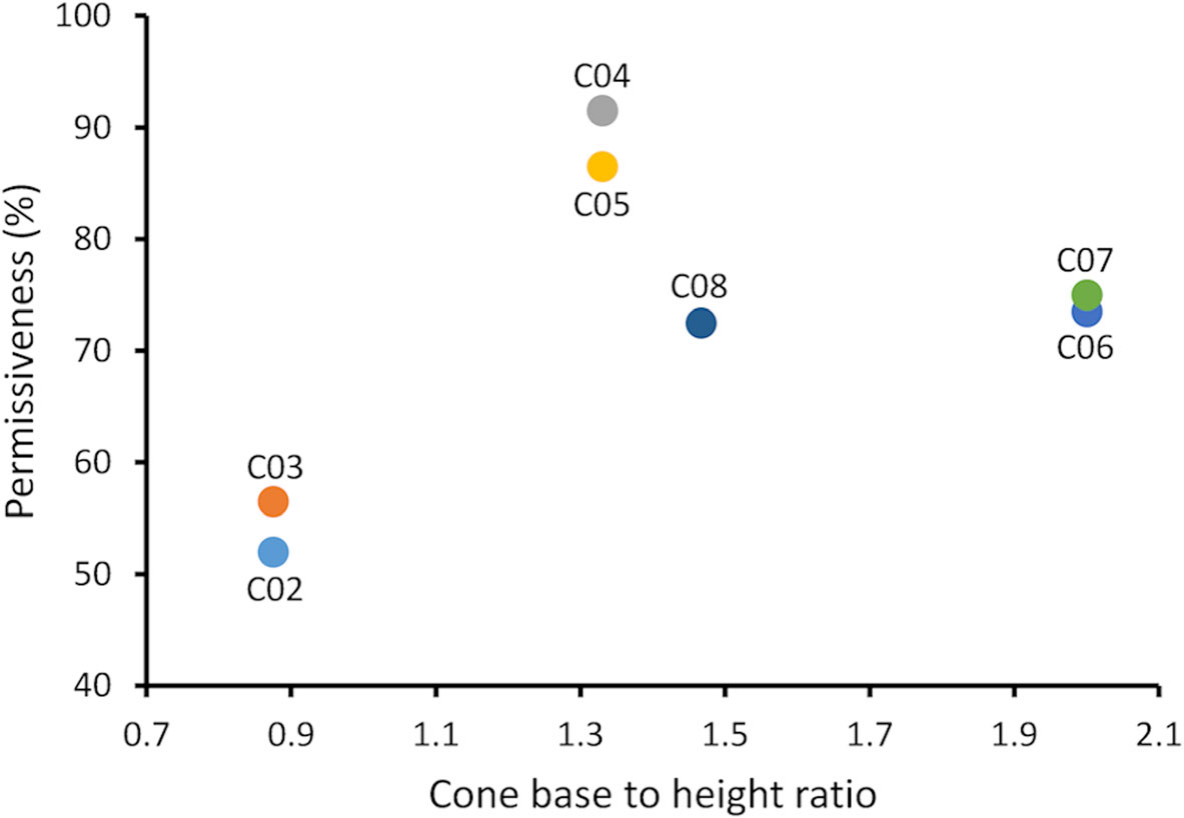
Effect of cone base-to-height ratio on the permissiveness of the permissive side of the cone-based screens

The fourth 3D screen design group was based on cone structures. In total, 12 different cone-based screens were constructed (C01-C12, Fig. 7a-l). They had varying cone base diameter, height, number, and screen material. C04 and C12 screens exhibited the highest (92%, 49/53) and lowest permissiveness (20%, 12/58) (Table 3) for the permissive sides within this group, respectively. All cone-based screens, however, had equally effective non-permissive sides as no mosquitoes were able to escape the double screen trap through the hole on the tip of the cones. Table 4 shows the number of mosquitoes used in each test. The C04 cone-based screen was also assigned the highest SP index (92) in the whole study. Moreover, further data analysis showed that the cone-based screens, C04 and C05, equipped with cones that had a base diameter to cone height ratio of 1.3 had the best performing permissive sides (Table 4). Ratios lower or higher than 1.3 decreased the permissiveness of the cones (Fig. 8).

## Discussion

No doubt exists that creating a physical barrier between mosquitoes and their host provides protection against mosquito-borne diseases. House screening, as a physical barrier to prevent mosquitoes from entering houses, usually involves screening windows and doors with mosquito screens. The concept behind this study was to design a unidirectional screen, a 3D screen that could be added in front of the traditional mosquito screen to create a window double screen trap. Twenty-five screen designs were tested in a wind tunnel under experimental conditions that simulate the window double screening to achieve this goal. Among those designs, a screen utilizing cone structures, C04, showed the best performance. The cone base to height ratio and the material used to make the cones were the decisive factors controlling the performance of the cone-based screens. A ratio of 1.3 was optimal for obtaining better performance of cone-based screens, ratios below or above 1.3 appeared to decrease mosquito maneuvering ability to find the entry point on the permissive side of the screens. In addition, cone-based screens with a mesh size or material reducing airflow were less effective for allowing higher number of mosquitoes to pass through the permissive sides of the screens. This was probably due to blocking volatiles emanating from the lure. The second-best SP index was calculated for Cyl2, which had 9 mm diameter cylinders. The cylinder diameter was the decisive factor for the performance of these screens. A diameter of 12 mm was too wide, allowing mosquito passage from both sides, while a 7 mm diameter was too small, blocking most mosquito passage through both sides. The 9 mm diameter was, however, the right size to permit most mosquitoes to pass through the permissive side than through the non-permissive side. The decisive factor for the filament-based screen performance as a unidirectional screen was mesh size (equivalent to diameter size in the cylinder-based screens) and filament length. The bigger the mesh, the higher the number of mosquitoes passing through. Filament length exhibited a dichotomous role, as the longest filaments resulted in less mosquitoes passing through the permissive side, the side that did not expose the filaments, but enhanced passing through the side exposing them. The longest filaments as the first contact point appeared to enhance mosquito maneuvering ability to pass through the screen. In case of prism-based screens, the slit height and the pore size at the edge of the prisms were the decisive factors controlling the unidirectional screen efficiency.

The two best performing 3D screen designs, the cone-based (C04) and the prism-based (W5), shared similarities with the principles used in window exit traps already in use in research for decades. Window exit traps utilizing either a single funnel [15] or a single prism [16], however, have a much longer depth (30–35 cm) than the 3D screens (5 cm) designed in this study. The small depth of the 3D screens makes them more suitable for home use than window exit traps. Diabaté et al. [13] reported another entry trap, the Lehmann’s funnel, as a means to control mosquito population. The Diabaté et al. [13] design had an even longer depth (51 cm) than the aforementioned mosquito window traps, making the adoption of the design less favorable. Window double screen traps based on the 3D screens with small depth would require only minimum maintenance and are environment friendly. They are insecticide-free and require less material for construction. In addition, a 3D screen material needed to build a window double screen trap would only cost roughly $2, making it a cheap mosquito control method that would protect all sleepers in the house. Window double screens traps would be built of two parallel frames, connected at the top by hinges, holding the two screens. The trapped mosquitoes are removed by simply pulling the outside frame upwards using the connected knob. The 3D screen-based window double screen traps would also be a small modification to the traditional window screening known to various communities. The use of the 3D screen-based window trap would provide dual benefit, as it would act as both a traditional window screen and a mosquito trap. The optimal configuration of a window double screen trap would use two 3D screens opposite to each other, one on each side of the trap. When using this configuration, mosquitoes that entered the house through other openings would also be trapped when leaving the house. Trapping mosquitoes in this configuration would be particularly useful if mosquitoes would have gotten a blood meal from infected individuals living in the house.

Semi-field studies will be the next step to test the performance of the cone-based screens. Although the 3D cone-based screen showed a promising performance in the wind tunnel, its real-life performance remains to be determined. This is because mosquito host-seeking behaviour in the wind tunnel was activated in a manner different from the natural field conditions. This was primarily due to the absence of carbon dioxide, the most important sensory cue, from the wind tunnel setup. Additionally, the wind tunnel is a closed compartment, while houses, or even experimental huts, in the field have eaves and other openings that allow carbon dioxide and body odours to diffuse and make these openings the main entry points for mosquitoes. Therefore, the experimental design of the future semi-field work will tackle these two issues by including test conditions with closed or open eaves. In other test conditions, the cone-based 3D screens will be installed opposite to each other on both sides of the window to allow trapping mosquitoes that would have entered through eaves and trying to escape through the windows. The performance of the 3D screen in the field studies will be then determined based on the analysis of the various test conditions.

Reports on the association between reducing the number of mosquitoes in houses and protection from malaria are scarce. Nevertheless, a literature review by Lindsay et al. [10], including pioneering work from the end of the nineteenth century, presented some evidence that house screening was associated with protection against malaria transmission, infection and morbidity [10]. Moreover, Tusting et al. [17] conducted a meta-analysis on interventional and observational studies published from 1900 to 2013 to assess whether improved housing was associated with reduced exposure to infectious bites and malaria infection. This work revealed that house screening is an important factor for reducing the risk for malaria. The most significant stand-alone evidence obtained so far, however, is from a three-armed randomized controlled trial conducted in The Gambia [12]. This trial measured the clinical outcomes of house screening in an African setting and found that window and door screens and closed eaves halved the prevalence of anemia in children [12]. Other studies have also demonstrated the importance of house screening or blocking mosquito entry points on reducing the number of mosquitoes inside houses [10, 11, 18–23]. Eave screening to prevent entry of mosquitoes was shown to protect households from exposure, not only to malaria vectors but also to vectors of lymphatic filariasis and Rift Valley fever and O’nyong nyong viruses [18]. Installation of an extra ceiling (plywood, synthetic netting, insecticide-treated synthetic netting or plastic insect screen) below the eaves level resulted in a significant reduction in *Anopheles gambiae* and *Mansonia* spp. collected in the experimental huts compared to control huts without the extra ceilings [11]. In a similar study, a ceiling made of local papyrus mats fitted below eave level also reduced significantly *A. gambiae* and *A. funestus* mosquito densities in treated houses [19]. A more recent work conducted in Chano, Ethiopia, also showed a significant reduction in indoor density of *A*. *arabiensis* by screening windows and doors with metal meshes, and closing openings on eaves and walls by mud [21]. A study in Sri Lanka also showed that better constructed houses (brick and plaster walls and tiled roofs) led to a significantly lower malaria incidence rate than houses that were poorly constructed (mud or cadjan walls and cadjan-thatched roofs) [22]. In the latter study, well-built houses also harbored significantly lower numbers of indoor-resting mosquitoes than the poorly constructed houses. In a more recent study conducted in Mozambique, covering gables and eaves with locally available materials significantly reduced the number of *A. funestus* and *A. gambiae* entering houses [23].

## Conclusions

Considering the available literature on the benefits of house screening, little doubt exists that 3D screen-based double screen traps fitted on windows, doors, or eaves could provide protection against malaria. The 3D screen-based window double screen traps would behave similarly to the traditional mosquito screens. In addition, when used on a large scale they could provide an extra benefit by reducing the number of mosquitoes and blood-engorged mosquitoes (in the two 3D screens configuration-based trap) within a given community. Nevertheless, it remains to be determined how efficient the 3D screen-based double screen traps are in capturing mosquitoes under experimental field conditions.

## Additional files

Additional file 1: Figure S1. Spacer mesh fabric made by Baltex, UK. (TIFF 367 kb)

Additional file 2: Figure S2. Higher resolution images of the designed and tested 3D mosquito screens. (DOCX 7797 kb)

## Abbreviations

ACTs: Artemisinin-based combination therapies
C01-C12: Cone-based screens 1–12
Cyl1-Cyl3: Cylinder-based screens 1–3
IRS: Indoor residual spraying of insecticides
ITNs: Insecticide-treated bed nets
S4-S8: Filament-based screens 1–5
W1-W5: Prism-based screens 1–5
