# Supplementary figures and images for: 3D mosquito screens to create window double screen traps for mosquito control
Source: Parasit Vectors. 2017 Aug 29;10:400. doi: 10.1186/s13071-017-2322-2 (PMC5576366; doi:10.1186/s13071-017-2322-2)

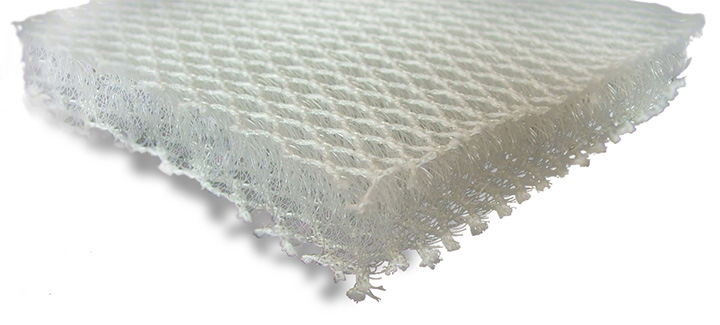

Supplement: Supplementary file 1 — Spacer mesh fabric made by Baltex, UK. (TIFF 367 kb) [file 13071_2017_2322_MOESM1_ESM.tif]
