# Supplementary material for: 3D mosquito screens to create window double screen traps for mosquito control
Source: Parasit Vectors. 2017 Aug 29;10:400. doi: 10.1186/s13071-017-2322-2 (PMC5576366; doi:10.1186/s13071-017-2322-2)
Supplement: Supplementary file 2 — Higher resolution images of the designed and tested 3D mosquito screens. (DOCX 7797 kb) [file 13071_2017_2322_MOESM2_ESM.docx]

**Additional file 2**

**Figure S2.** Larger images of the 3D screens designed and tested throughout the study. Panels 1-25 show individual screens. Left side of the panel shows the non-permissive side of the screen while the right side shows the permissive side of the screen. Panel number corresponds to screen number listed on Table 1 in the main text.

| 1. Cyl1  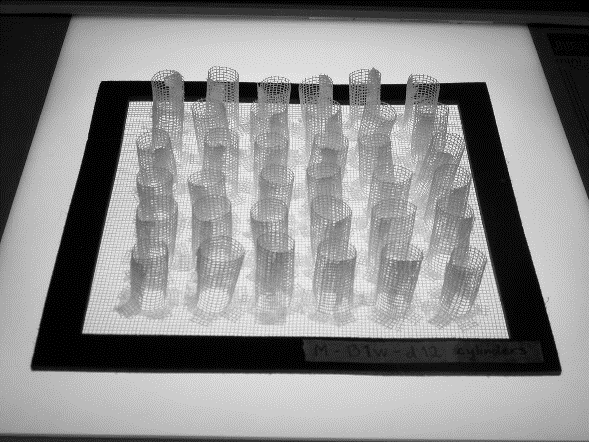 | 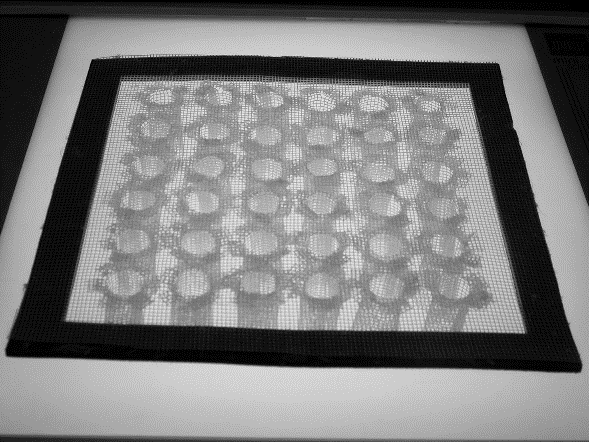 |
| --- | --- |
| 2. Cyl2  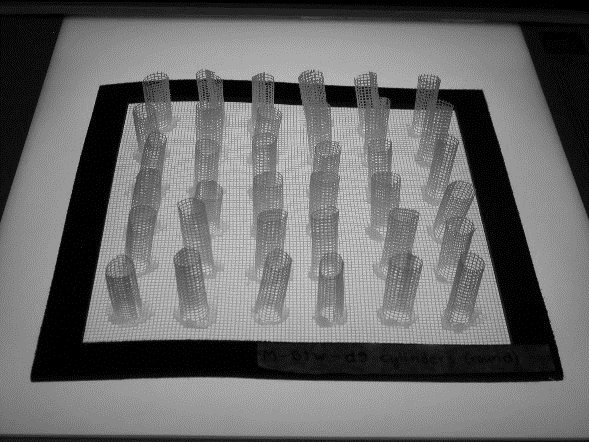 | 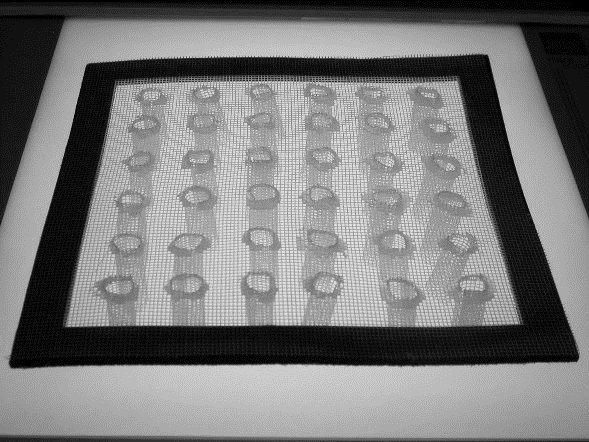 |
| 3. Cyl3  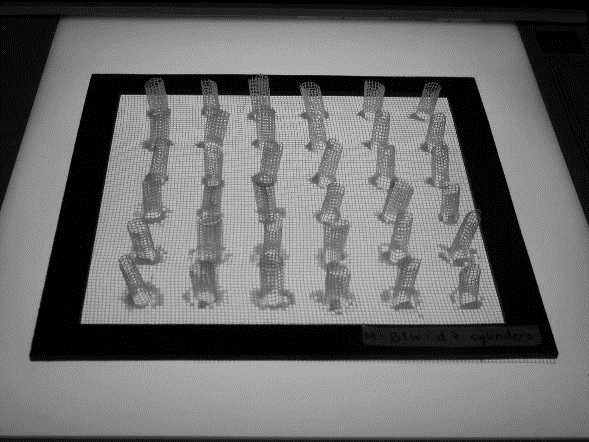 | 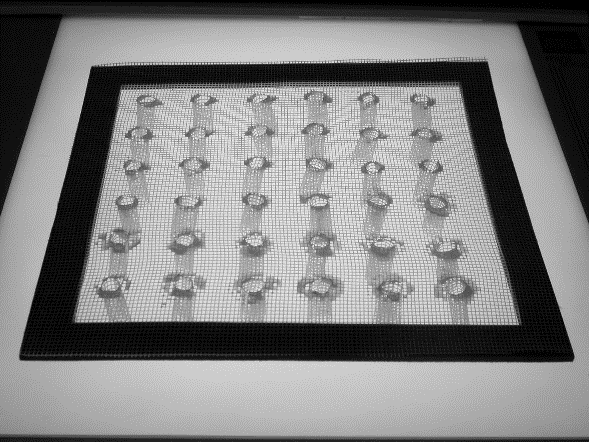 |
| 4. S4  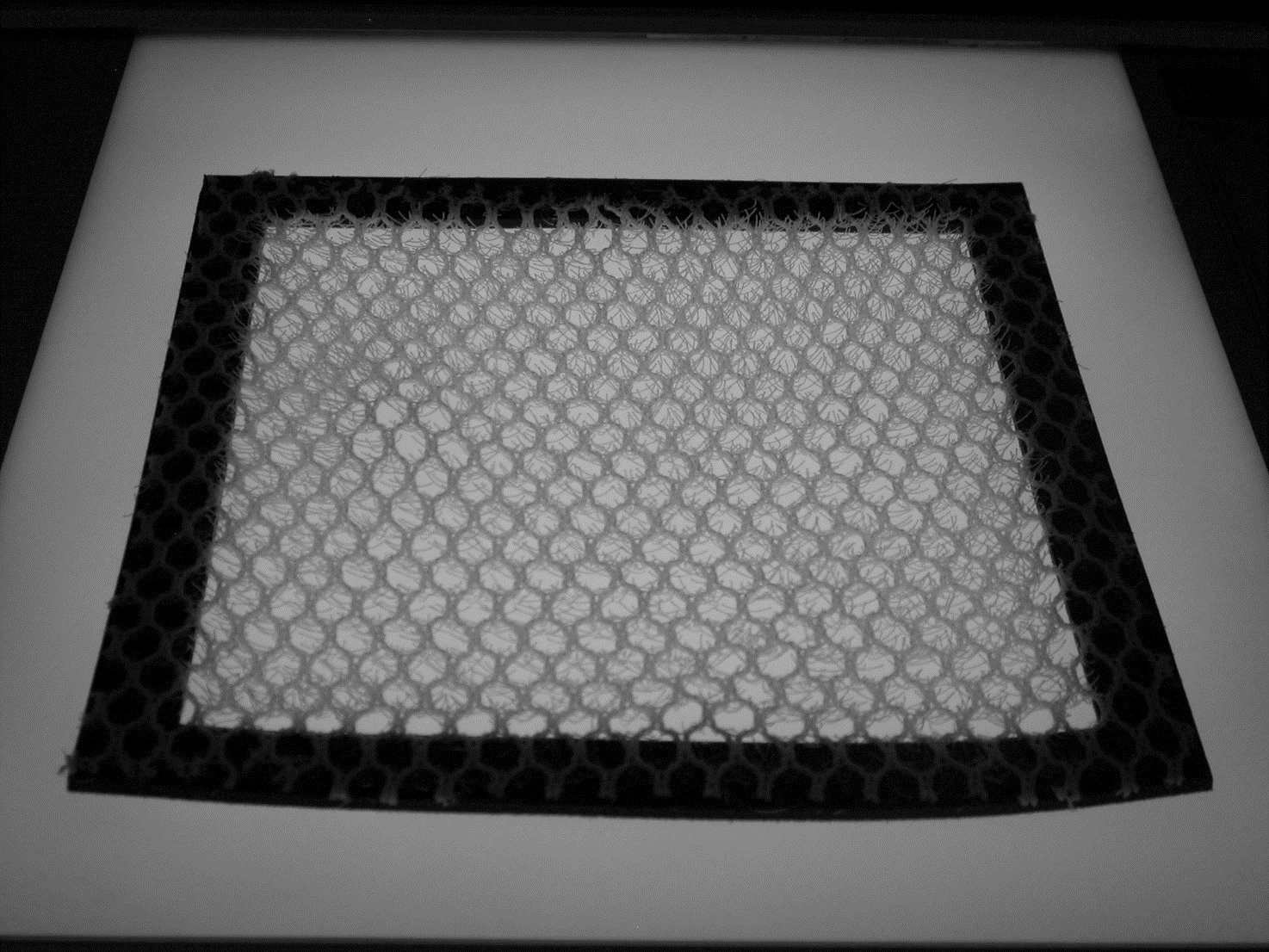 | 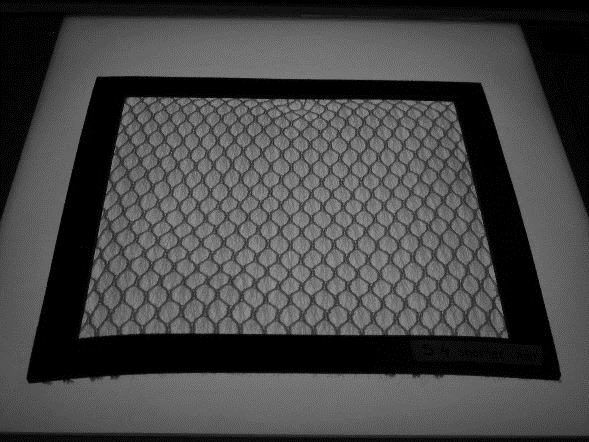 |
| 5. S5  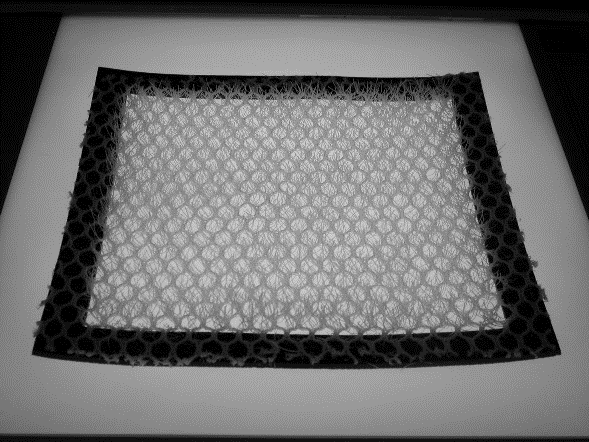 | 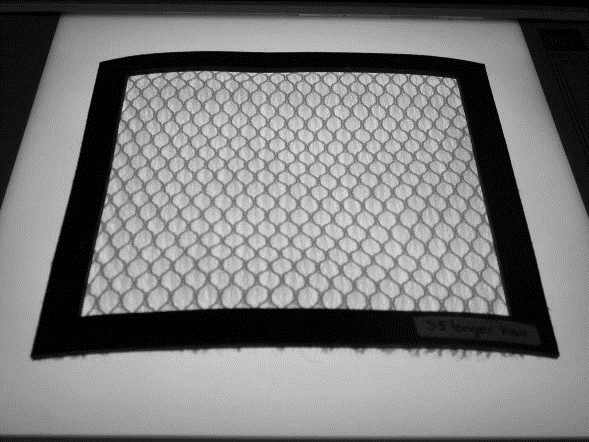 |
| 6. S6  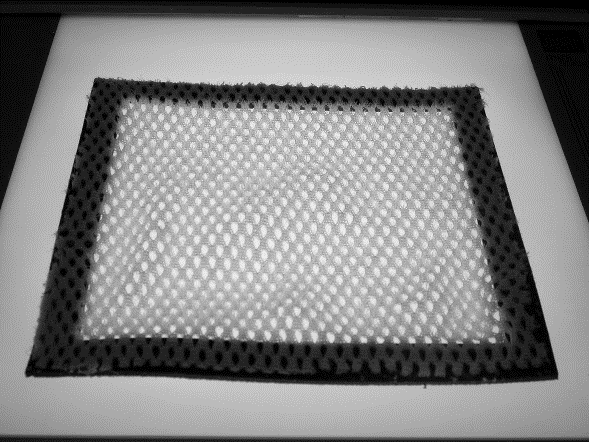 | 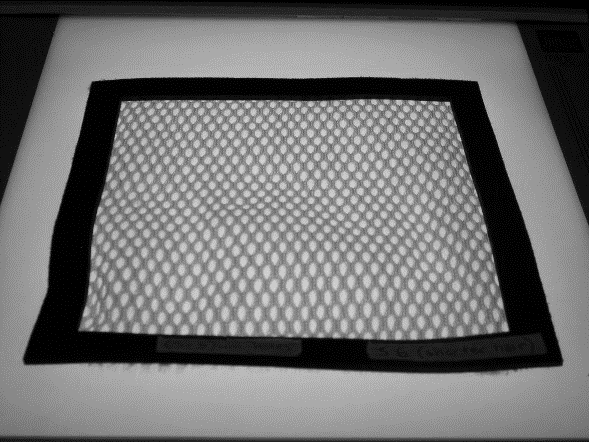 |
| 7. S7  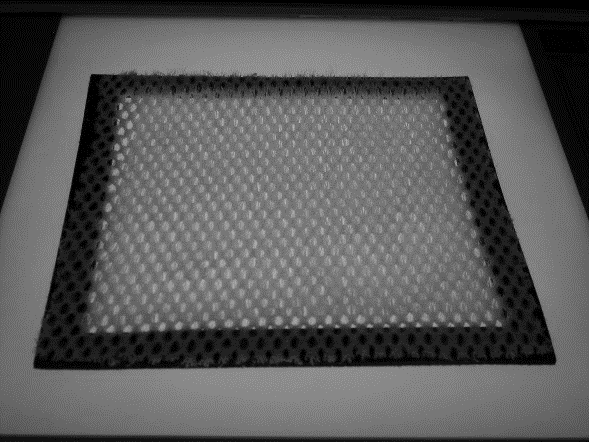 | 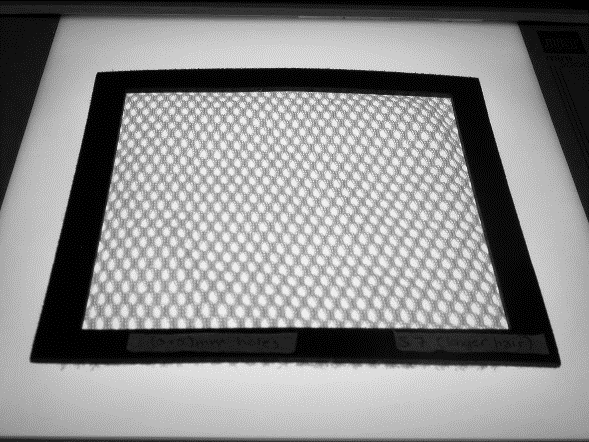 |
| 8. S8  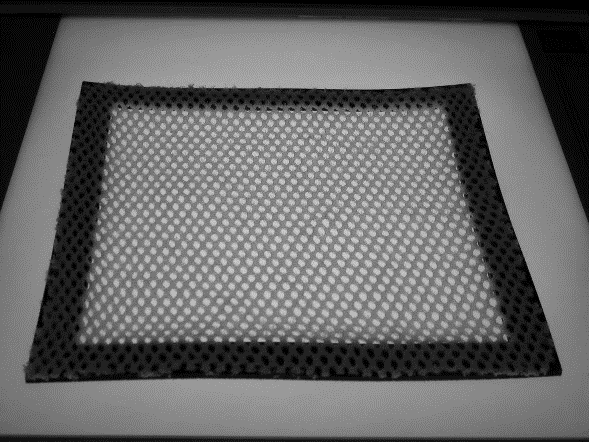 | 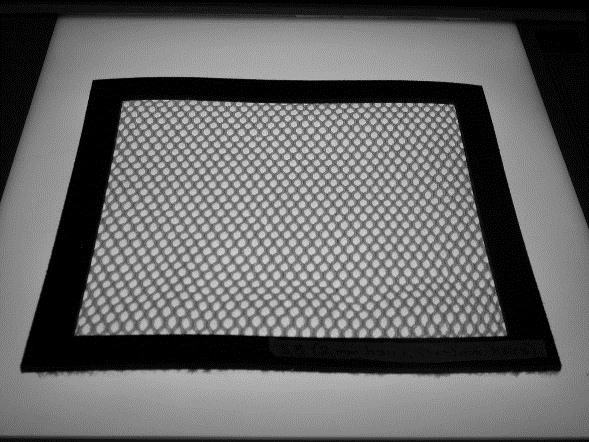 |
| 9. W1  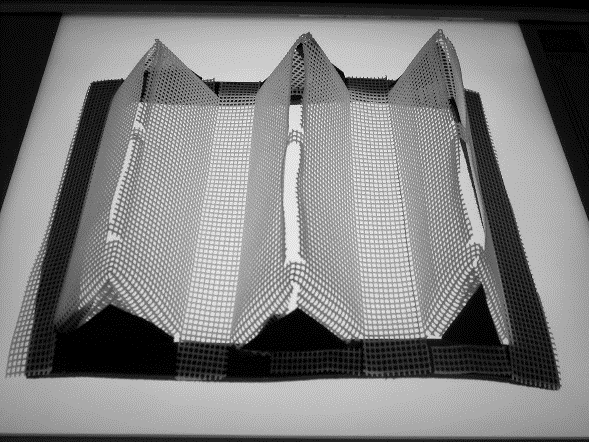 | 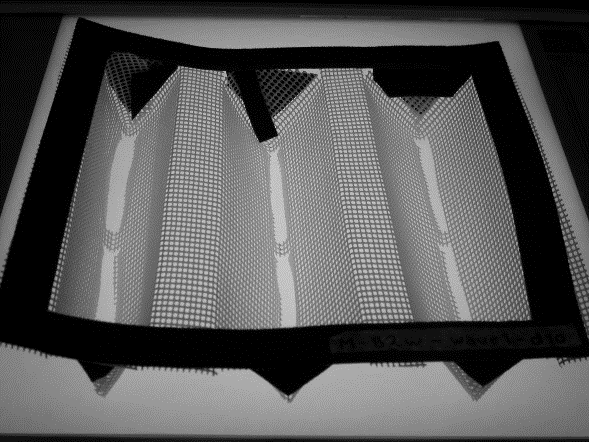 |
| 10. W2  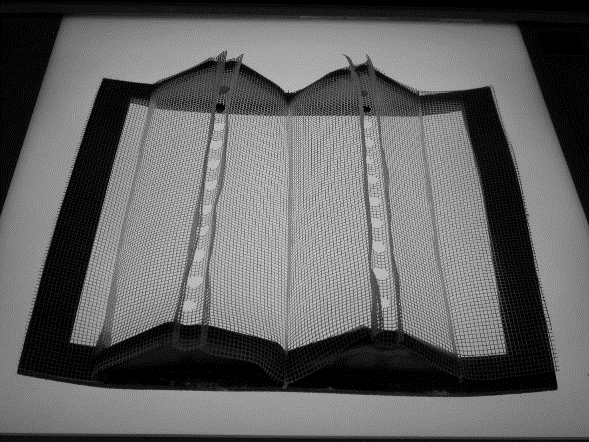 | 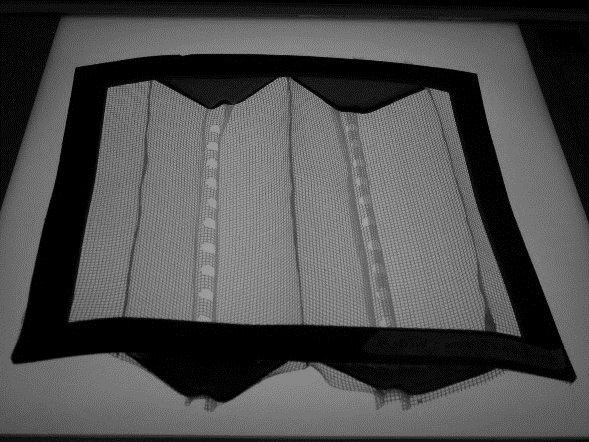 |
| 11. W3  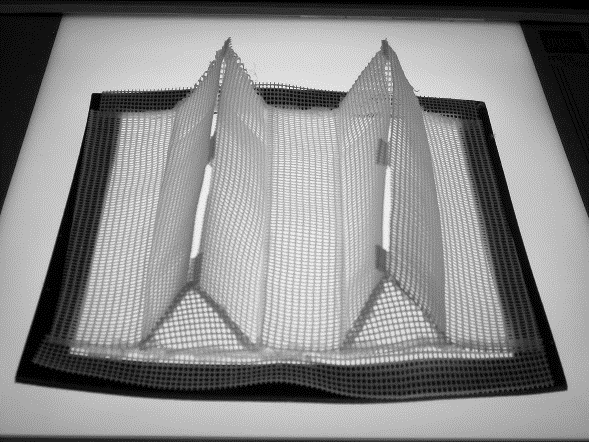 | 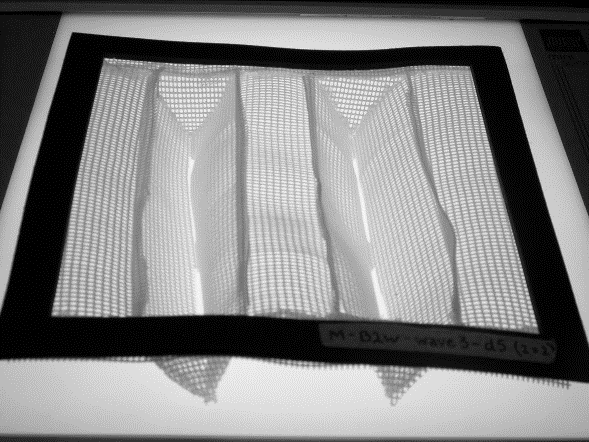 |
| 12. W4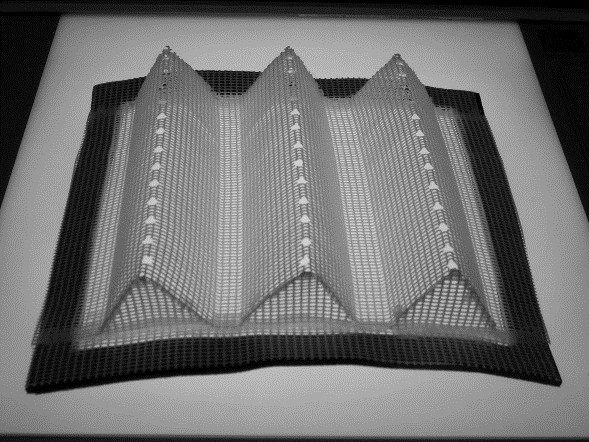 | 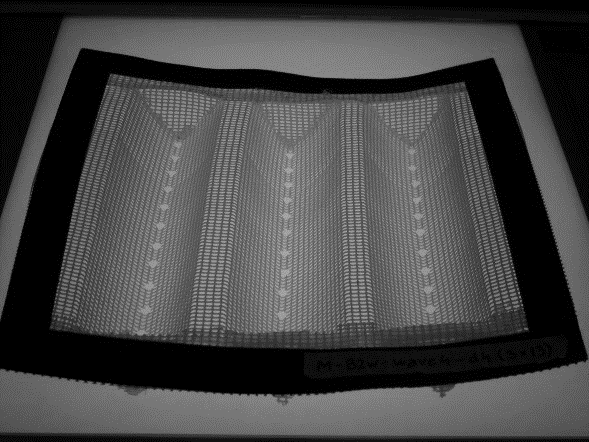 |
| 13. W5  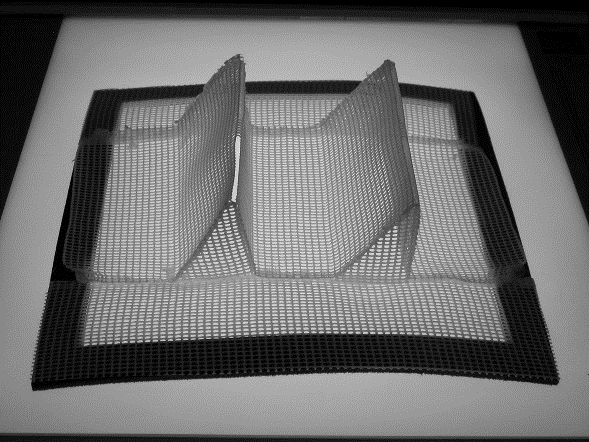 | 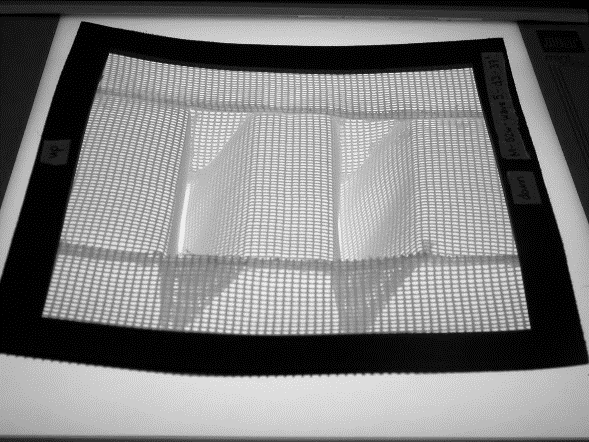 |
| 14. C01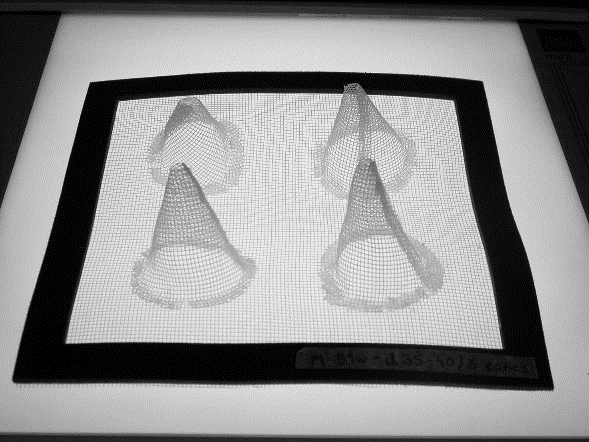 | 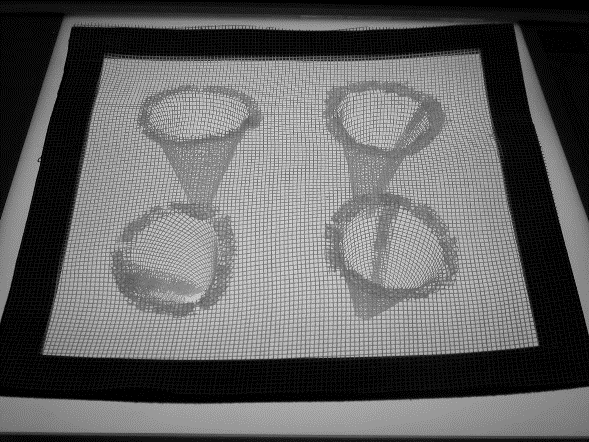 |
| 15. C02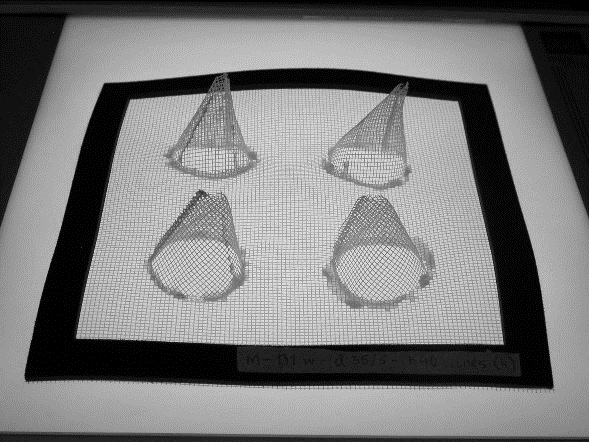 | 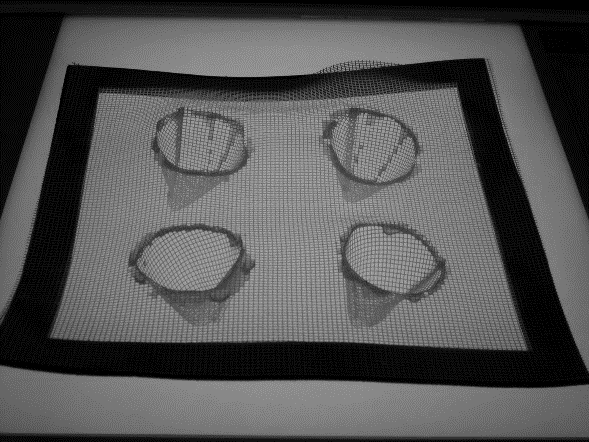 |
| 16. C03  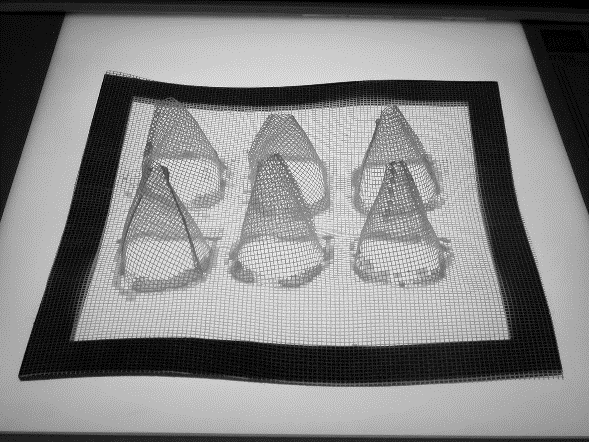 | 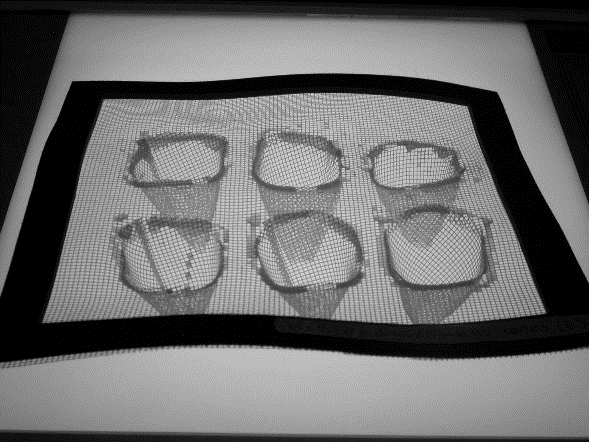 |
| 17. C04  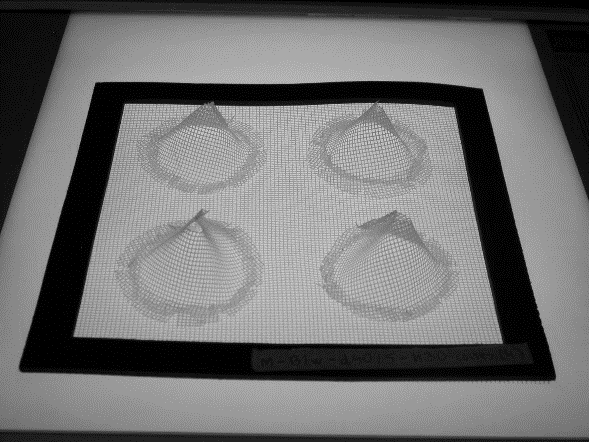 | 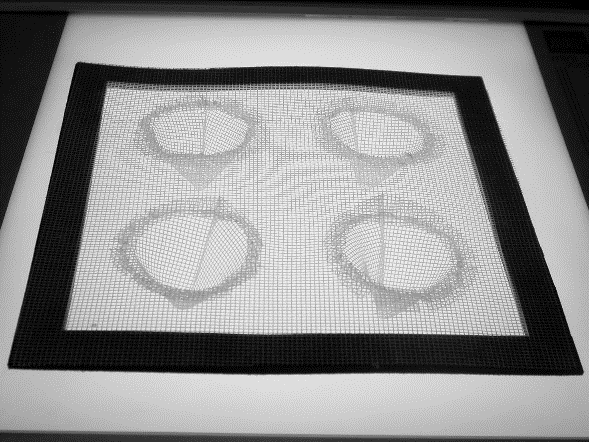 |
| 18. C05  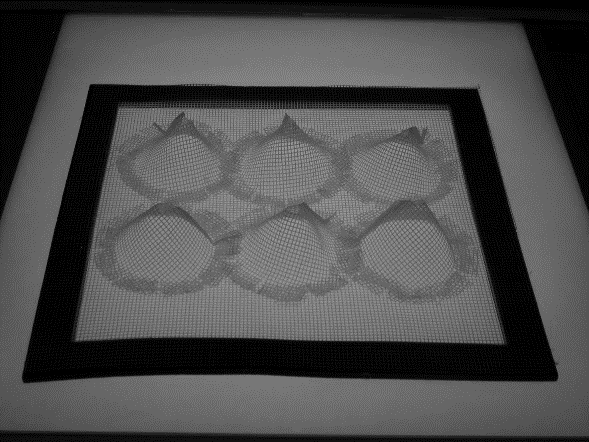 | 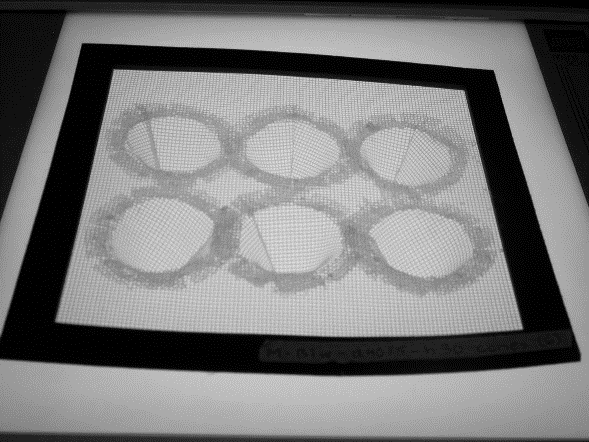 |
| 19. C06  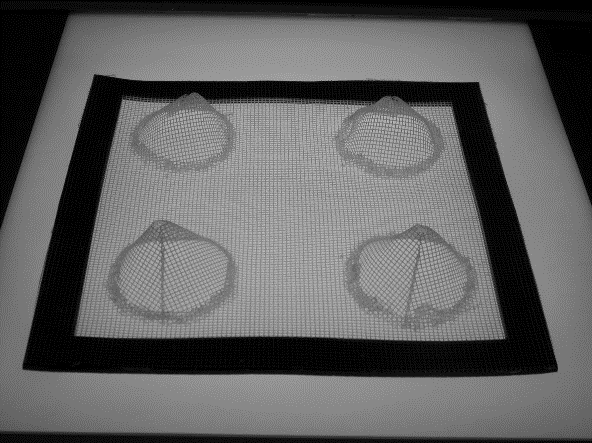 | 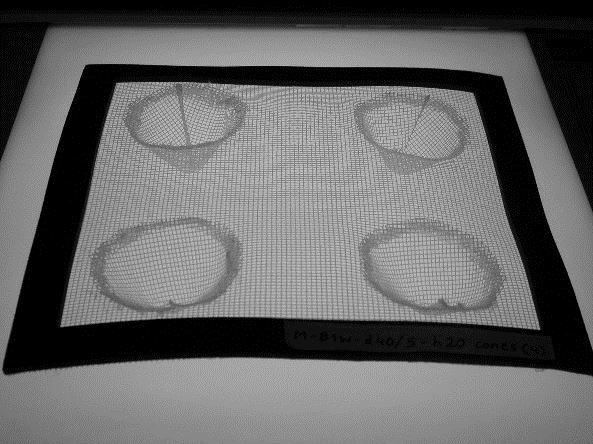 |
| 20. C07  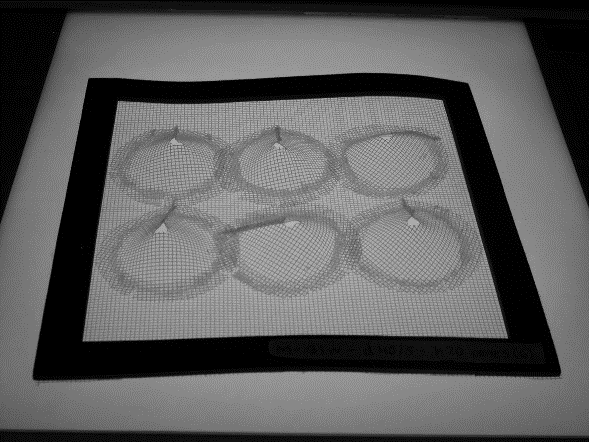 | 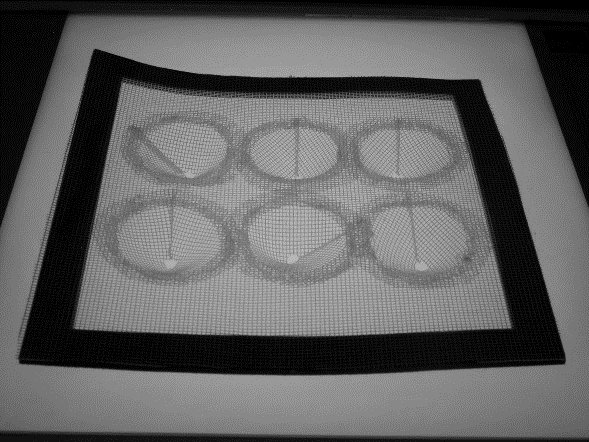 |
| 21. C08  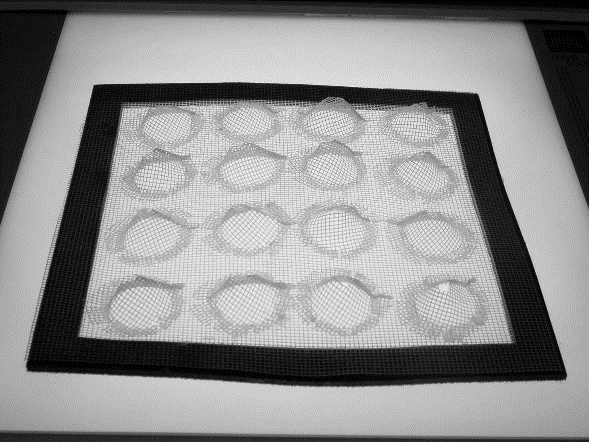 | 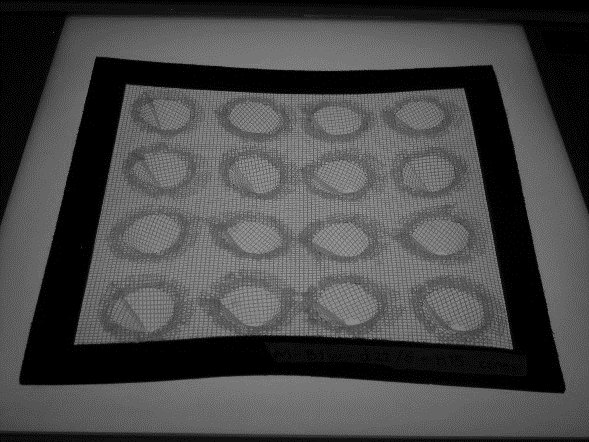 |
| 22. C09  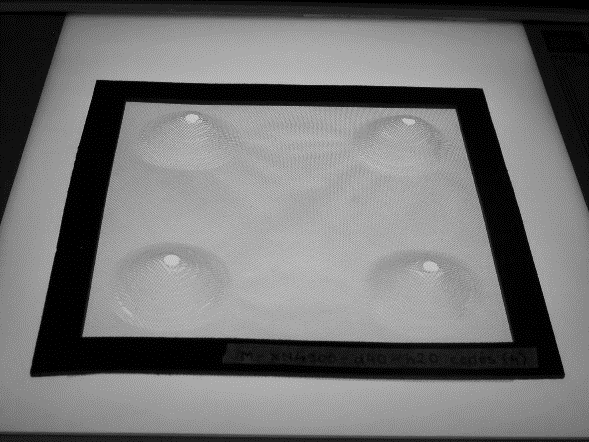 | 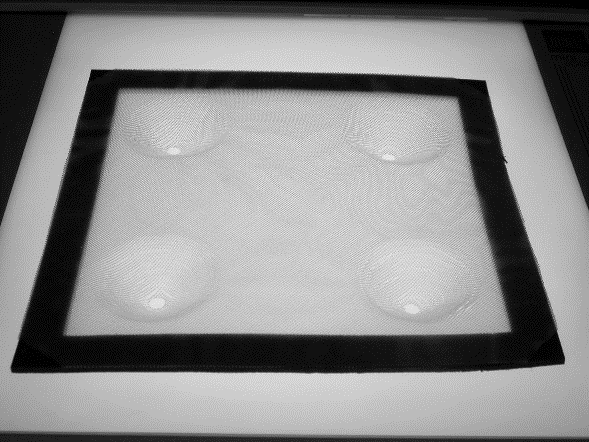 |
| 23. C10  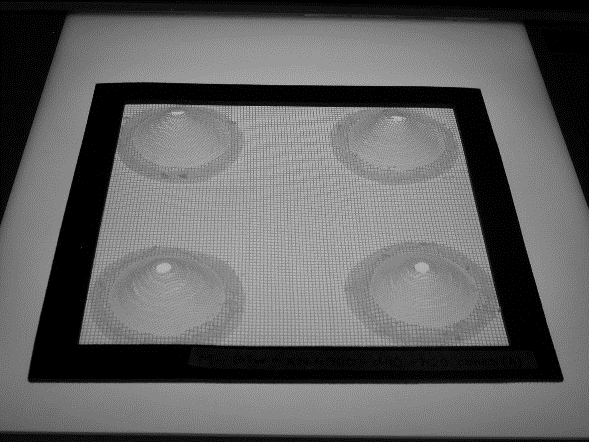 | 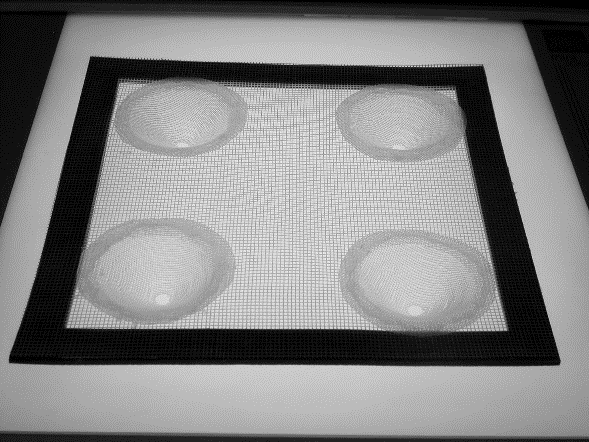 |
| 24. C11  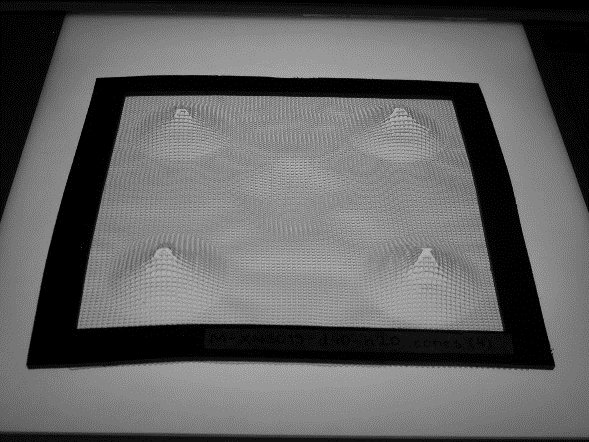 | 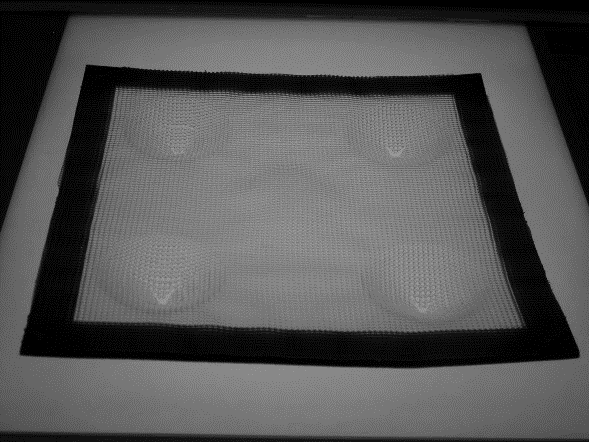 |
| 25. C12  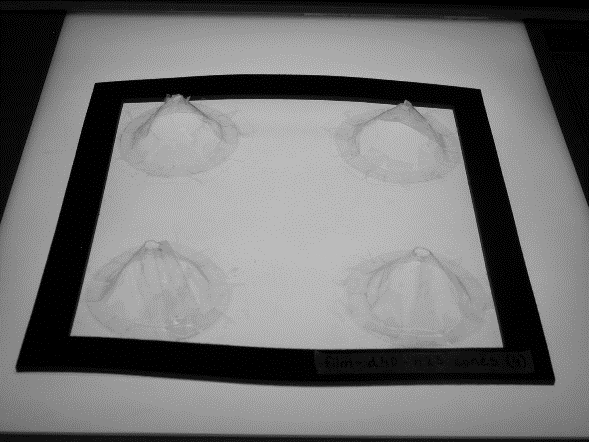 | 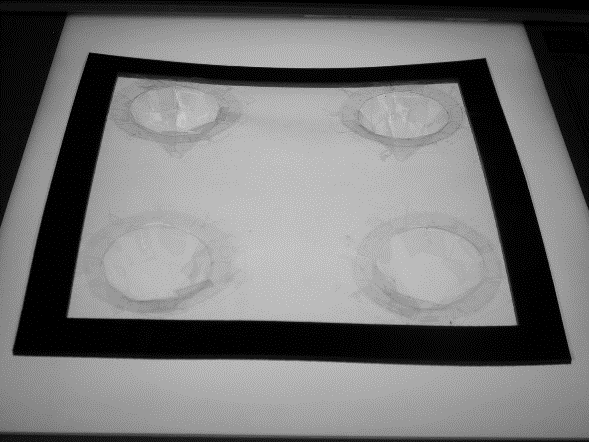 |
